# A Mixed Mirror-image DNA/RNA Aptamer Inhibits Glucagon and Acutely Improves Glucose Tolerance in Models of Type 1 and Type 2 Diabetes*

**DOI:** 10.1074/jbc.M112.444414

**Published:** 2013-06-06

**Authors:** Axel Vater, Simone Sell, Przemyslaw Kaczmarek, Christian Maasch, Klaus Buchner, Ewa Pruszynska-Oszmalek, Pawel Kolodziejski, Werner G. Purschke, Krzysztof W. Nowak, Mathias Z. Strowski, Sven Klussmann

**Affiliations:** From the ‡ NOXXON Pharma AG, Max-Dohrn-Strasse 8–10, 10589 Berlin, Germany,; the § Department of Animal Physiology and Biochemistry, Poznan University of Life Sciences, 35 Wolynska Street, 60637 Poznan, Poland, and; the ¶ Department of Hepatology and Gastroenterology and Interdisciplinary Centre of Metabolism: Endocrinology, Diabetes and Metabolism, Charité-Universitätsmedizin Berlin, Augustenburger Platz 1, 13353 Berlin, Germany

**Keywords:** Aptamers, Diabetes, DNA, Drug Design, Drug Discovery, Spiegelmer, Glucagon Inhibitor, Mirror-image Aptamer, Type 1 Diabetes, Type 2 Diabetes

## Abstract

Excessive secretion of glucagon, a functional insulin antagonist, significantly contributes to hyperglycemia in type 1 and type 2 diabetes. Accordingly, immunoneutralization of glucagon or genetic deletion of the glucagon receptor improved glucose homeostasis in animal models of diabetes. Despite this strong evidence, agents that selectively interfere with endogenous glucagon have not been implemented in clinical practice yet. We report the discovery of mirror-image DNA-aptamers (Spiegelmer®) that bind and inhibit glucagon. The affinity of the best binding DNA oligonucleotide was remarkably increased (>25-fold) by the introduction of oxygen atoms at selected 2′-positions through deoxyribo- to ribonucleotide exchanges resulting in a mixed DNA/RNA-Spiegelmer (NOX-G15) that binds glucagon with a *K_d_* of 3 nm. NOX-G15 shows no cross-reactivity with related peptides such as glucagon-like peptide-1, glucagon-like peptide-2, gastric-inhibitory peptide, and prepro-vasoactive intestinal peptide. *In vitro*, NOX-G15 inhibits glucagon-stimulated cAMP production in CHO cells overexpressing the human glucagon receptor with an IC_50_ of 3.4 nm. A single injection of NOX-G15 ameliorated glucose excursions in intraperitoneal glucose tolerance tests in mice with streptozotocin-induced (type 1) diabetes and in a non-genetic mouse model of type 2 diabetes. In conclusion, the data suggest NOX-G15 as a therapeutic candidate with the potential to acutely attenuate hyperglycemia in type 1 and type 2 diabetes.

## Teaser

**Background:** An increased glucagon/insulin ratio is known to contribute to hyperglycemia in diabetes.

**Results:** NOX-G15, a mirror-image mixed DNA/RNA glucagon-neutralizing aptamer, was identified. It improved glucose tolerance in models of type 1 and 2 diabetes.

**Conclusion:** NOX-G15 may be useful for treatment of type 1 and 2 diabetes.

**Significance:** The new therapeutic candidate may help to reduce insulin need in diabetes.

## Introduction

Glucagon and insulin are two counter-regulatory pancreatic hormones that participate in controlling glucose homeostasis. The principal function of glucagon is the stimulation of hepatic glucose production and output in the fasting state. Compatible with this notion, glucagon levels in the fasting state are increased, whereas ingestion of carbohydrate-rich food causes a fall of plasma glucagon levels with the concomitant reduction of endogenous glucose production (1).

More than 40 years ago Unger (2) observed that the glucagon/insulin ratio is consistently increased in type 2 diabetes mellitus (T2DM).^2^ Later Unger and Orci (3) proposed a bihormonal hypothesis to explain the development of hyperglycemia in this disease: patients with T2DM have an impaired insulin response and an inadequately suppressed or even increased glucagon secretion following ingestion of a carbohydrate-rich meal compared with non-diabetic subjects. Although the lack of insulin-dependent suppression of glucagon release from pancreatic α-cells in advanced T2DM contributes to hyperglucagonemia (4), the exact mechanisms explaining the dysfunctional behavior of pancreatic α-cells are still not entirely known. Certainly, hyperglucagonemia in T2DM aggravates hyperglycemia due to persistent stimulation of hepatic glucose production (5).

The role of glucagon in the pathophysiology of experimentally induced type 1 diabetes mellitus (T1DM) was also recently reported by Unger and colleagues (6, 7). Deletion of the glucagon receptor in mice resulted in protection from streptozotocin (STZ)-induced diabetes (6) and severe hyperglycemia appeared when the knock-out was reverted by adenoviral glucagon receptor expression (7). Taking into account that the α-cell mass is relatively and/or absolutely increased in both T1DM and T2DM, which is reflected by uncontrolled glucagon release, the principle of lowering glucagon seems to be a promising strategy for therapeutic intervention.

Numerous attempts have been undertaken by the pharmaceutical industry to develop potent small molecule glucagon receptor antagonists or antibodies for the clinical use (extensively reviewed by Refs. 1 and 8). None has gained marketing approval thus far, mainly due to toxicity or lack of selectivity. However, an antisense oligonucleotide (ISIS-GCGR_Rx_) targeting the mRNA of the glucagon receptor is currently in Phase I clinical development (9) and a glucagon receptor antagonist (LY-2409021) has entered Phase II, being the most advanced compound to date (10). Despite these recent developments the identification of novel substances selectively inhibiting glucagon action is still of great therapeutic interest due to the possibility of direct interference with the crucial pathophysiological mechanisms of hyperglycemia in diabetes.

Spiegelmer®^3^ are functional mirror-image oligonucleotide scaffolds that can bind and inhibit target molecules comparable with protein-based scaffolds like antibodies. To identify a Spiegelmer, at first libraries of ∼10^15^ different oligonucleotide sequences made from natural nucleotides in the d-configuration are screened for variants that can bind to the non-natural configuration (mirror-image form) of an intended target molecule (*e.g.* a d-polypeptide). Those oligonucleotides that bind to the selection target (so-called *aptamers* (11)) are enriched through cycles of selection and enzymatic amplification (SELEX process (12)). At the end of the *in vitro* selection process, the enriched aptamers are singled-out by cloning, sequenced, and individually analyzed for their affinity to the mirror-image selection target. Next, selected aptamer sequences are made from mirror-image building blocks (l-nucleotides) and the resulting Spiegelmers show identical binding to the target in the natural configuration (*e.g.* l-polypeptide) (13). The non-natural chirality makes Spiegelmers resistant to RNases and DNases that are prevalent in biological fluids (14). Several Spiegelmers have been generated against bioactive molecules and have shown proof of concept *in vivo* (15–17). Three Spiegelmer-based drug candidates directed against other targets are currently in Phase II clinical development and have proven so far to be safe, well tolerated, and non-immunogenic (18).

Here, we describe the discovery of a DNA-based anti-glucagon Spiegelmer that was further improved in affinity by exchanging single deoxynucleotides to ribonucleotides. The resulting candidate, NOX-G15, binds and neutralizes glucagon with high specificity and an affinity in the low nanomolar range. NOX-G15 has no measurable affinity to related peptides such as glucagon-like peptide (GLP)-1, GLP-2, prepro-vasoactive intestinal peptide (prepro-VIP), and glucose-dependent insulinotropic peptide (GIP); the exception, however, is oxyntomodulin (OXM) that contains the full 29-amino acid sequence of glucagon followed by an 8-amino acid long carboxyl-terminal extension. In experimental non-genetic mouse models of T1DM and T2DM (6, 19, 20) NOX-G15 acutely improved glucose tolerance after an intraperitoneal glucose challenge. Thus, NOX-G15 potentially offers a novel and alternative mode of treatment of hyperglucagonemia-induced hyperglycemia in T1DM and T2DM.

## EXPERIMENTAL PROCEDURES

### 

#### 

##### Peptides and Oligonucleotides

Glucagon (HSQGTFTSDYSKYLDSRRAQDFVQWLMNT) was synthesized as an all-d-peptide with and without a C-terminal biotin modification. Biotin was coupled via a TTDS linker (TTDS = 1,13-diamino-4,7,10-trioxatridecan-succinic acid) or a double PEG2 linker (PEG2 = 8-amino-3,6-dioxaoctanoic acid), respectively, to an ethylendiamino spacer at the peptides' C termini (Biosyntan, Berlin, Germany). Natural human l-glucagon and its C terminally biotinylated form (via PEG2-PEG2-ethylendiamino-linker) were purchased from Bachem (Bubendorf, Switzerland) and Biosyntan, respectively. Oligonucleotides were synthesized using controlled pore glass from Prime Synthesis (Ashton, PA) and standard phosphoramidite chemistry at NOXXON Pharma (Berlin, Germany). l-Phosphoramidites were from ChemGenes Corp. (Wilmington, MA), Transgenomic (Paisley, Scotland, UK), and Innovasynth (Khopoli, Maharashtra, India). d-Phosphoramidites were from Thermo Fisher (Milwaukee, WI) and Proligo (Hamburg, Germany). The synthetic DNA library with 38 internal random positions had the sequence 5′-GAGGA TGCCT GTCAG GATGC ACT-N_38_-AGTG CTACG TTCAG ACACA TCC-3′, with N having an equal probability for each of the four nucleotides. During *in vitro* selection, the DNA library was amplified with the oligo(dT) primer 5′-TTTTT TTTTT TTTTT TTTTT XXGGA TGTGT CTGAA CGTAG C-3′ (X = triethylene glycol spacer, Glen Research, Sterling, VA) and the reverse primer 5′-GAGGA TGCCT GTCAG GATGC-3′. The use of these primers enables discrimination between the library sense strand (83 nt) and the antisense strand (103 nt) by denaturing polyacrylamide gel electrophoresis (PAGE), because thermostable DNA polymerases cannot read through XX during PCR leaving the oligo(dT) tail uncopied (21). For *in vivo* application the 5′-end of the glucagon-binding Spiegelmer l-257-E1-030-6xR (all-l-GCGGG_OH_ AAATG G_OH_GAG_OH_ G_OH_ GCTAG GTGGA_OH_ A_OH_GGAA TCTGA GCGC) was modified with an aminohexyl linker (American International Chemical Inc., Framingham, MA) and conjugated to 40-kDa polyethylene glycol (PEG, JenKem, Allen, TX) (22). The conjugate was designated NOX-G15. Spiegelmer concentrations and doses always refer to the oligonucleotide part as anhydrous free acid; PEG is not taken into account due to molecular weight distribution and slightly variable PEG size. A non-functional control Spiegelmer with the same nucleobase composition was generated by reversing the NOX-G15 sequence. The Spiegelmer was designated revNOX-G15 after 5′ PEGylation (PEG-aminohexyl-CGCGA GTCTA AGGA_OH_A_OH_ GGTGG ATCGG_OH_ G_OH_AGG_OH_G TAAAG_OH_ GGCG). To determine NOX-G15 plasma concentrations, a biotinylated reverse complementary DNA capture probe to the 3′-part of NOX-G15 (biotin-(hexaethylene glycol)_2_-5′-all-l-GCGCT CAGAT TCCTT CCACC-3′) was synthesized.

##### In Vitro Selection

DNA aptamers were selected by incubating the C terminally biotinylated d-glucagon with the single-stranded DNA library that had been 5′-labeled with [γ-^32^P]ATP by T4 polynucleotide kinase (Invitrogen, Darmstadt, Germany) in selection buffer (20 mm Tris, pH 7.4, 150 mm NaCl, 5 mm KCl, 1 mm MgCl_2,_ 1 mm CaCl_2_, 0.1% Tween 20, 0.1% CHAPS, 100 μg/ml of human serum albumin, 10 μg/ml of yeast RNA). The zwitterionic detergent CHAPS (Biomol, Hamburg, Germany) was included in the buffer to prevent the appearance of precipitates or gels resulting from glucagon fibrillation and amyloid formation (23, 24). In early rounds, the binding reactions were conducted in solution at 37 °C overnight and in later rounds for 1 to 3 h. Peptide-DNA complexes were immobilized on streptavidin or neutravidin coupled to agarose beads, high capacity agarose *plus* beads or high capacity streptavidin *ultra link plus* beads (Thermo Scientific, Rockford, IL), which were added to the reactions at the end of the incubation time. A negative selection step was introduced from round 3 on by preincubating the library with the immobilization matrix before adding the peptide to the binding reactions. After immobilization of glucagon-DNA complexes on the beads, the beads were washed by repeated re-suspension in selection buffer to partition non-binding or weak binding DNA molecules. The percentage of bound molecules was measured in a scintillation counter (LS6500; Beckman Coulter, Fullerton, CA) and triggered the stringency of the washing procedure as well as the choice of the d-glucagon concentration in the following selection round. Starting from initially 20 μm, the peptide concentration could gradually be decreased to 12.8 pm. The concentration of the DNA library was roughly adjusted in parallel to the peptide concentration, thus starting the selection process with a library consisting of ≈ 2.4 × 10^15^ molecules. Bound DNA was amplified directly on beads by PCR using Vent exo^−^ DNA polymerase (New England Biolabs, Ipswich, MA) or *Taq* DNA polymerase (Invitrogen). After strand separation by denaturing PAGE the sense strand was labeled and used for the following selection round. Because of the stagnating selection progress in round 12 the DNA libraries for rounds 13, 16, 19, 23, and 26 were amplified by mutagenic PCR to close gaps in the sequence space around aptamers in the already enriched library thus enabling the selection of more glucagon-affine sequences. For this a first PCR containing the nucleotide analogs 8-oxo-dGTP and dPTP (Jena Bioscience, Jena, Germany) in addition to the conventional dNTPs (25) was performed for 16–18 cycles. This was followed by a second amplification starting with 1/100 of the first PCR product in the presence of manganese chloride and increased magnesium concentrations (26) for a similar number of cycles. DNA obtained from round 26 was cloned and sequenced.

##### Post-selection Optimization

After identification of the most affine DNA Spiegelmer sequence, l-257-E1-001, we tested whether chemical backbone modifications would increase its affinity. For this, individual l-deoxyribonucleotides were exchanged for their l-ribonucleotide analogs (A → A_OH_; C → C_OH_; G → G_OH_; T → U), because these are the only commercially available alternative l-nucleotides. The variants were analyzed for binding using a competitive pull-down assay with labeled unmodified Spiegelmer l-257-E1-001 and an excess of each individual mutant as competitor. Those ribonucleotide positions that resulted in better binding were combined and analyzed for synergistic effects.

##### Determination of Affinity to Glucagon Using Competitive Pull-down Assays

The affinity of Spiegelmers to glucagon was measured in a pull-down assay format at 37 °C by competing the Spiegelmer binding to biotinylated glucagon with non-biotinylated glucagon. Through addition of two d-configured A nucleotides at the 5′-end, the Spiegelmer could be labeled with ^32^P using T4 polynucleotide kinase. Concentrations of 0.1–0.2 nm were incubated with a constant amount of biotinylated glucagon and varying concentrations of non-biotinylated glucagon as competitor at 37 °C in selection buffer for 2–3 h. The time was set to allow for complete equilibration at low concentrations. Peptide-Spiegelmer complexes were immobilized on neutravidin-agarose *plus* beads that were kept suspended for 20 min by intermittent mixing. The concentration of biotinylated glucagon was adjusted to achieve, without competition, around 10% Spiegelmer binding to the biotinylated peptide after immobilization and washing, to enable highly sensitive measurements of the dissociation constant *K_d_*_(comp)_. After washing the beads and removing the supernatant, radioactivity on the beads was determined in a scintillation counter. The fraction bound (in %) was plotted against the concentration of non-biotinylated glucagon. The dissociation constant *K_d_*_(comp)_ was obtained for each Spiegelmer by using the software GRAFIT (Erithacus Software, Surrey, UK) assuming a 1:1 stoichiometry.

##### Determination of Affinity to Glucagon and Related Peptides Using Surface Plasmon Resonance (Biacore)

Biotinylated human l-glucagon (500–600 response units) was immobilized on a neutravidin-coated carboxydextran (CM5) chip. The reference flow cell was equally loaded with neutravidin and subsequently blocked with biotin. All reagents for Biacore measurements were purchased from GE Healthcare, unless otherwise specified.

Kinetic parameters and dissociation constants were evaluated by a series of Spiegelmer injections at concentrations of 1000, 500, 250, 125, 62.5, 31.3, 15.6, 7.80, 3.90, 1.95, 0.980, and 0 nm in running buffer (20 mm Tris-HCl, pH 7.4, 150 mm NaCl, 5 mm KCl, 1 mm CaCl_2_, 1 mm MgCl_2_). At least one concentration was injected twice to monitor regeneration efficiency and flow cell integrity. All experiments were performed at 37 °C and a flow of 30 μl/min. The assay was double referenced, whereas the reference flow cell served as surface control (bulk contribution of each Spiegelmer concentration) and a series of buffer injections without analyte determined the bulk contribution of the buffer itself on both flow cells. Data analysis and calculation of dissociation constants (*K_d_*) was done with the BIAevaluation 3.1.1 software (BIACORE AB, Uppsala, Sweden) using a Langmuir 1:1 stoichiometric fitting algorithm, with a constant refractive index and mass transfer evaluation with an initial mass transport coefficient *k_t_* of 1 × 10^7^ (response units/M × s).

To address the selectivity of the Spiegelmer binding to glucagon-related peptides, a competitive Biacore assay was set up. Immobilization of biotinylated human glucagon was performed as described above. The Spiegelmer was injected at a fixed concentration of 125 nm together with a concentration series (2000, 1000, 500, 250, and 0 nm) of non-biotinylated competitors such as GLP-1(7–37), GLP-2(1–33), GIP, OXM, prepro-VIP(81–122), and glucagon itself. Each injection was repeated three times. The response units at the end of the injection period, after 360 s, of injection were determined and plotted using Prism 5.04 software (GraphPad, La Jolla, CA).

##### Inhibition of Glucagon-induced cAMP Generation in a Cell-based Assay

A stably transfected cell line expressing the human receptor for glucagon was generated by cloning the sequence coding for the human glucagon receptor (NCBI accession NM_000160) into a pCR3.1 vector (Invitrogen). CHO-K1 cells, adapted to growth in serum-free *UltraCHO* medium (Lonza, Basel, Switzerland), were transfected with the glucagon receptor plasmid; stably transfected cells were selected by treatment with geneticin. These cells expressing the glucagon receptor were plated on a 96-well plate (cell culture treated, flat bottom) at a density of 4–6 × 10^4^/well and cultivated in *UltraCHO* medium containing 100 units/ml of penicillin, 100 μg/ml of streptomycin, and 0.5 mg/ml of geneticin at 37 °C in 5% CO_2_ overnight. 20 min before stimulation, a solution of 3-isobutyl-1-methylxanthine was added to a final concentration of 1 mm.

Solutions of glucagon and various concentrations of Spiegelmer were made up in Hank's balanced salt solution, containing 1 mg/ml of BSA, and were incubated at 37 °C for 30 min. Shortly before addition to the cells, 3-isobutyl-1-methylxanthine was added to a final concentration of 1 mm.

After the growth medium had been removed, the solutions containing 0.5 nm glucagon and increasing Spiegelmer concentrations were added in triplicates to the cells and incubated at 37 °C for 30 min. The solutions were removed and the cells were lysed in lysis buffer, which is a component of the cAMP-Screen^TM^ System kit (Applied Biosystems, Darmstadt, Germany). This kit was used for determination of the intracellular cAMP content according to the supplier's instructions. The half-maximal inhibition of cAMP production (IC_50_) was calculated using nonlinear regression (four parameters fit) with Prism 5.04 software.

##### Pharmacokinetics of NOX-G15 in Mice

Plasma levels of NOX-G15 were determined in female NMRI mice after a single intraperitoneal injection of 10 mg/kg of NOX-G15 (16 mice per group). Each mouse was bled two times evenly across the study period, which equals 4 mice per time point. Blood samples were taken at 10 min, 1, 3, 8, 24, 48, and 96 h after dosing and lithium-heparin plasma was prepared. Dosing and blood sampling were performed by Heidelberg Pharma (Heidelberg, Germany). All animal procedures were approved by the local ethical committee and performed in accordance with the national guidelines for the care and use of animals in biomedical research.

##### Quantification of NOX-G15 in Plasma

NOX-G15 plasma levels were quantified by Biacore measurement using a biotinylated l-DNA hybridization probe, which is complementary to the 3′-part of NOX-G15 and was immobilized on a neutravidin-coated CM5 chip (GE Healthcare). An *N*-hydroxysuccinimide/1-ethyl-3-(3-dimethylaminopropyl)carbodiimide-activated and ethanolamine-blocked flow cell and a neutravidin-coupled biotin-blocked flow cell on the same sensor chip served as surface controls.

The thawed plasma samples were briefly vortexed, incubated on blue-ice for 5 min, and subsequently centrifuged at 13,000 × *g* for 5 min. Standards and plasma samples to be analyzed were injected in triplicate and the NOX-G15 concentration in unknown samples was calculated using a standard curve.

Association and dissociation of the binding event was recorded under optimized buffer conditions at 37 °C for 360 and 240 s, respectively. Regeneration was performed by injecting 15 μl of glycine-HCl, pH 1.5, and 15 μl of 50 mm NaOH. Every tenth analysis cycle, a NOX-G15 standard was injected to corroborate the in-use stability of the immobilized hybridization probe. All assays were double-referenced, as described above.

Data analysis was performed with the BIAevaluation 3.1.1 software. The association constant *k_a_* is defined as [1/M × s] thus the initial slope (response units/s) directly correlates with the concentration of the analyte.

The lower limit of quantification was 0.65 nm NOX-G15. Calculation of plasma half-life *t*½ was performed on the basis of a linear fitting algorithm of the NOX-G15 plasma elimination phase from 8 to 96 h after intraperitoneal injection.

##### Experimental Induction of T1DM

Male BALB/c mice were housed under standard conditions for several weeks before starting the experiment. STZ-induced diabetes was induced according to the protocol by Lee and colleagues (6). In brief, mice (31.3 ± 1.9 g of body weight) were first injected with STZ (100 mg/kg body weight) 3 weeks prior to receiving test substances followed by a second injection of STZ (80 mg/kg body weight) 2 weeks before the treatment. Development of the diabetic phenotype was monitored measuring fasting glucose levels (using a Roche Accu Check Active device, range 10–600 mg/dl) in 5 μl of blood taken from the tail tip (Fig. 7*A*). Serum levels of insulin after 2.5 h fasting were determined in a separate, equally treated cohort to avoid any influence on the intraperitoneal glucose tolerance test (ipGTT) results by drawing a high volume of blood. As expected, insulin levels after 2.5 h of fasting were strongly reduced in T1DM mice treated with this protocol (0.39 ± 0.21 ng/ml, *n* = 12) compared with healthy control mice (2.13 ± 0.58 ng/ml, *n* = 9) and resulting in severe fasting hyperglycemia (Fig. 7, *A* and *B*). In addition, body weight changes were also measured. Animals with a weight loss >25% compared with the initial body weight and animals with fasting blood glucose levels below 200 mg/dl or above 500 mg/dl were excluded from the study (Fig. 8, *A–C*). These criteria allowed inclusion of 35 animals. Animals (7 per group) were matched by body weight 1 day before the ipGTT (Fig. 8, *D* and *E*). The animal protocol was approved by the Local Ethical Commission for Investigation on Animals, Poznań University of Life Sciences (permission number 18/2011).

##### Experimental Induction of T2DM

Male BALB/c mice weighing 27.5 ± 3.5 g were fed *ad libitum* a high-fat diet (HFD) containing 60% kcal from fat (D12492, Research Diets Inc.) for 10 weeks. After 8 weeks of HFD feeding, a single dose of STZ (100 mg/kg body weight) was administered to induce alterations of blood glucose and serum insulin levels mimicking late-stage T2DM (27). Induction of T2DM was confirmed by increased fasting blood glucose levels. Mice from a separate cohort with HFD/STZ-induced diabetes mellitus had 2.5 h fasting serum insulin of 2.89 ± 0.11 ng/ml (*n* = 11), being similar to the values of non-diabetic mice 2.13 ± 0.19 ng/ml (*n* = 9) (Fig. 7, *A* and *B*). In addition body weight changes were monitored. Fig. 10, *A* and *B,* show the course of blood glucose and body weight. Mice with fasting blood glucose below 200 or above 300 mg/dl were excluded from the study. Likewise, mice that did not have a stable body weight profile before and 1 week after the STZ injection despite HFD feeding were excluded. Animals (8–9 mice per group) were matched by body weight 1 day before the start of the ipGTT (Fig. 10, *C* and *D*). The animal protocol was approved by the Local Ethical Commission for Investigation on Animals, Poznań University of Life Sciences (permission number 18/2011).

##### Intraperitoneal Glucose Tolerance Test

After 2.5 h of fasting, an aqueous solution of the test compound (1 or 10 mg/kg of NOX-G15), the reference peptidyl glucagon antagonist (2 or 4 mg/kg of des-His^1^-Glu^9^-GCG) (28, 29), or vehicle was injected intraperitoneally. The glucose tolerance test was started by injecting mice pretreated with test agents with glucose (2 g/kg, intraperitoneally) at a time point *t* = 0 min, 90 min after compound administration. Glucose was dissolved in 0.9% NaCl.

Blood glucose measurements were done both before test compound administration and before glucose administration. Further blood glucose measurements were done at 20, 40, 70, and 100 min post-glucose administration. After sacrificing the animals 100 μl of blood was drawn to determine Spiegelmer plasma levels. To determine whether the observed effects were glucagon-specific, an additional ipGTT including revNOX-G15 was conducted in a new cohort of T1DM mice. Experimental parameters like diabetes induction, inclusion and randomization criteria were unchanged with the exception of the blood glucose determination that was done at: 5, 15, 30, 45, 90, and 120 min. AUC_glucose_ determination was therefore run from 0 to 120 min.

##### Statistical Analysis

Differences between treatment groups were analyzed for significance using one-way analysis of variance (ANOVA) and Tukey post-test analysis.

## RESULTS

### 

#### 

##### Identification of Glucagon-binding DNA Oligonucleotides

**FIGURE 1.**
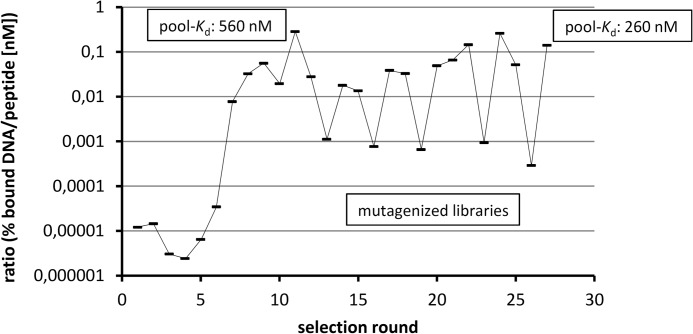
**Course of the *in vitro* selection.** As a measure for the enrichment of biotinylated d-glucagon-binding DNA sequences, the ratio of the fraction of library bound to the target immobilized on streptavidin or neutravidin beads *versus* the applied biotinylated peptide (d-glucagon) concentration is plotted against the selection round numbers. A steep increase in binding is visible in rounds 6 to 8 followed by stagnation in rounds 9 to 12. In the following rounds the regular alternation between mutagenic and standard PCR results in alternating ratios.

**FIGURE 2.**
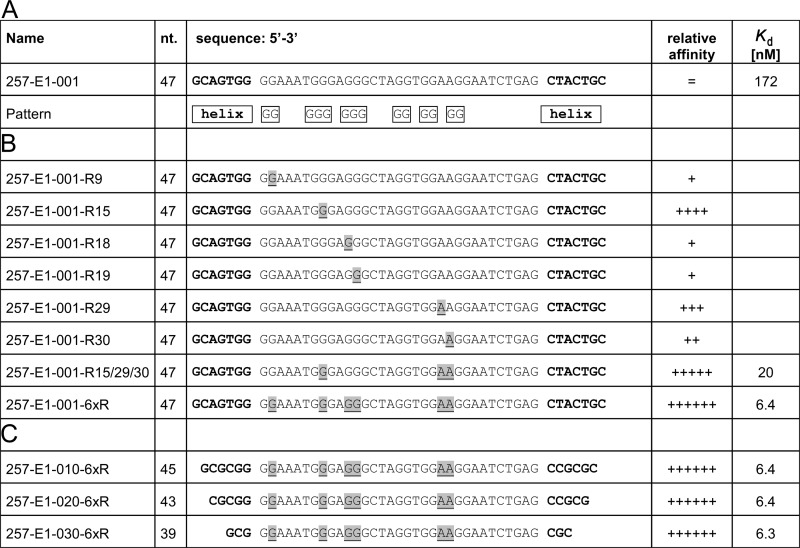
**Optimization of the glucagon-binding Spiegelmer.** *A,* original sequence from *in vitro* selection without primer binding sites. *B,* variants with deoxyribonucleotide to ribonucleotide exchanges; *underline* = ribonucleotide. *C*, truncation steps to the minimal binding sequence.

The *in vitro* selection process was started in the first round with 20 μm biotinylated d-glucagon and a single-stranded DNA library at a 1:1 ratio in solution. Bound DNA molecules were partitioned using streptavidin-coated beads via the biotin modification of the d-glucagon and amplified by PCR. In the course of the *in vitro* selection the stringency was increased by decreasing both target and DNA concentration (single-stranded DNA, obtained through strand separation after PCR). As shown in Fig. 1, a marked increase in binding became visible in rounds 6 to 8 without further improvement through round 12. The affinity of the enriched DNA library was determined to be 560 nm after round 11. To further increase the pool affinity, mutagenic PCR in combination with reduced stringency was applied in regular alternation to standard PCR followed by high stringency selection steps. Selection rounds with mutagenized libraries are characterized by a marked decrease of the ratio between binding percentage and applied peptide concentration, whereas selection rounds with libraries amplified by standard PCR show a sharp increase of this ratio indicating a fast recovery of affinity that was lost by the preceding mutagenesis (Fig. 1). At the end of the selection process, a target-binding library fraction was still detectable at a 12.8 pm peptide concentration in the equilibrium binding reaction. Of note, because the selection target is concentrated by the streptavidin beads for separation, the local concentration of biotinylated d-glucagon on the beads after target immobilization is higher than in solution. The library affinity after round 26 was determined to be 260 nm. Thus, an affinity gain of approximately a factor of two was achieved through the mutagenic selection strategy between rounds 13 and 26. The DNA from round 26 was cloned and sequenced. Alignment revealed only one family of highly identical sequences that differed just by point mutations. By omitting most of the primer binding sites, a set of truncated aptamers (47 nt) was synthesized, and individual sequences were ranked using the labeled library after round 26 as a reference. The sequence of the best aptamer (257-E1-001) is depicted in Fig. 2*A*. Striking elements in the primary structure are the self-complementary termini that may form a helix of 7 basepairs, and a pattern of two triple and four double G repeats that may be involved in a G-quadruplex motif (30). In a pull-down assay, the affinity of this aptamer to d-glucagon was 137 nm. The aptamer was synthesized as a Spiegelmer by using mirror-image (l-configured) nucleotides. l-257-E1-001 inhibited glucagon-induced cAMP formation in CHO cells expressing the glucagon receptor with an IC_50_ of ≈ 200 nm (data not shown).

##### Post-selection Optimization

To improve the binding affinity of the lead Spiegelmer l-257-E1-001 to glucagon, it was modified by exchanging l-DNA to corresponding l-RNA nucleotides, which are the only alternative mirror image nucleic acid building blocks that are commercially available for solid phase oligonucleotide synthesis. All variants synthesized were ranked in a competitive pull-down assay using Spiegelmer l-257-E1-001 as reference. The majority of the ribose-modified Spiegelmers showed a decreased or unchanged affinity. However, modifications at nucleotide positions 9, 15, 18, 19, 29, and 30, counted from the 5′ end, improved the affinity to glucagon (Fig. 2*B*). The exchange at position 15 resulted in the highest increase in affinity, followed by positions 29 and 30. The other positive modifications only showed a modest improvement. Therefore, at first the most promising three modifications were combined in the Spiegelmer l-257-E1-001-R15/29/30. The Spiegelmer showed a further improvement compared with the previously best molecule 257-E1-001-R15 and was determined to possess a *K_d_*_(comp)_ of 20 nm. Combination of all six affinity-enhancing modifications (l-257-E1-001-6xR) improved the *K_d_*_(comp)_ to 6.4 nm (Fig. 2*B*). Finally, eight nucleotides of variant l-257-E1-001-6xR could be truncated at the termini by experimental helix stabilization to result in the candidate l-257-E1-030-6xR (*K_d_*_(comp)_ = 6.3 nm) with a length of 39 nt (Fig. 2*C*).

**FIGURE 3.**
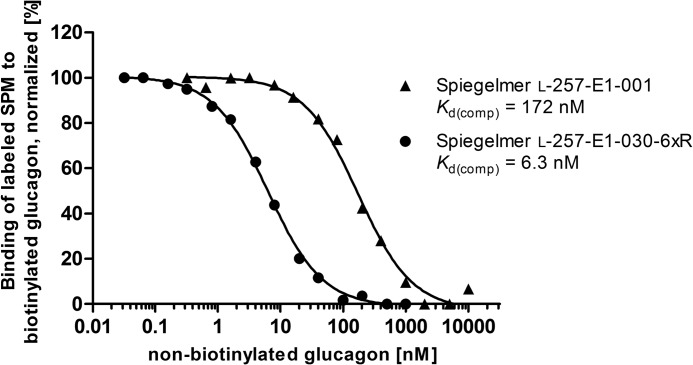
**Determination of the dissociation constants of the l-glucagon-binding lead Spiegelmer (SPM) before (▴) and after (●) optimization in a competitive pull-down assay.**

In the competitive pull-down assay the dissociation constant of the truncated lead candidate l-257-E1-030-6xR to l-glucagon was thus 27 times better than the *K_d_* of the original Spiegelmer l-257-E1-001 (172 nm, Fig. 3). The Spiegelmer l-257-E1-030-6xR was then PEGylated at its 5′-end and designated NOX-G15 after verification that PEGylation did not alter affinity by Biacore.

##### In Vitro Characterization of NOX-G15 Using Biacore-based Selectivity Analysis

**FIGURE 4.**
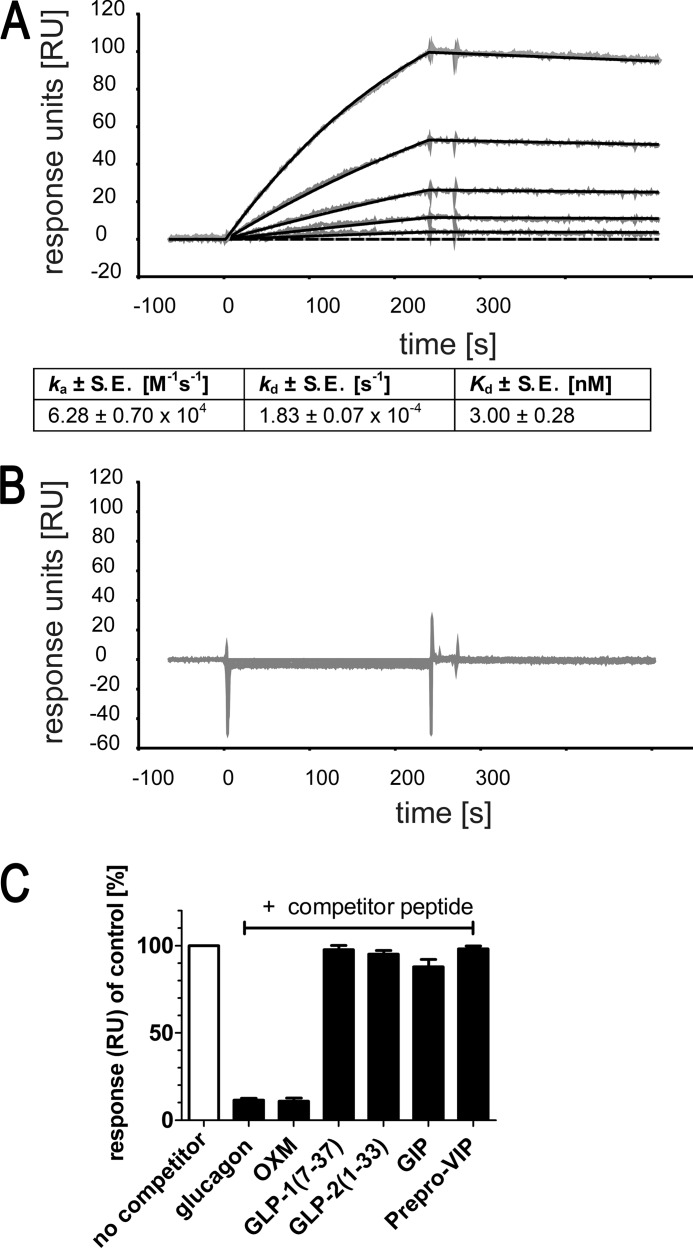
**Biacore-based affinity and selectivity analysis of NOX-G15.** *A,* representative Biacore sensorgram of NOX-G15 injected at 15.6, 7.80, 3.90, 1.95, 0.980, and 0 nm over immobilized l-glucagon. The equilibrium dissociation constant (*K_d_*) of NOX-G15 binding to l-glucagon was deduced by fitting the association and dissociation phases of these concentrations in 4 independent experiments. *B*, sensorgram of the reverse control Spiegelmer, revNOX-G15, injected at 1000, 500, 250, 125, 62.5, 31.3, 15.6, 7.8, 3.9, 1.95, 0.980, and 0 nm, over immobilized l-glucagon on the same chip as NOX-G15 measurement. *C,* NOX-G15 binding to immobilized l-glucagon without competitor (*control*) was normalized to 100%. When NOX-G15 is co-injected with glucagon or related peptides (competitor peptides), NOX-G15 association to immobilized glucagon is reduced if binding to the soluble competitor occurs (responses shown only for 2000 nm competitor peptides).

Using surface plasmon resonance, the dissociation constant of the PEGylated Spiegelmer NOX-G15 to l-glucagon was determined to be 3.00 ± 0.28 nm (mean ± S.E., *n* = 4), whereas the control Spiegelmer revNOX-G15 did not bind to l-glucagon (Fig. 4, *A* and *B*).

The cross-reactivity of NOX-G15 to glucagon-related peptides was analyzed in a competitive binding assay with immobilized l-glucagon by co-injection of NOX-G15 and increasing concentrations of a competitor peptide. NOX-G15 showed comparable binding to glucagon (self-competition) and OXM. Because OXM consists of the native glucagon sequence plus 8 amino acids at the C terminus, this result indicates that the C terminus of glucagon is not part of the epitope that is bound by the Spiegelmer. The other peptides related to glucagon, *i.e.* GLP-1(7–37), GLP-2(1–33), prepro-VIP(81–122), and GIP were not recognized by NOX-G15 (Fig. 4*C*).

##### Cell-based Assay

**FIGURE 5.**
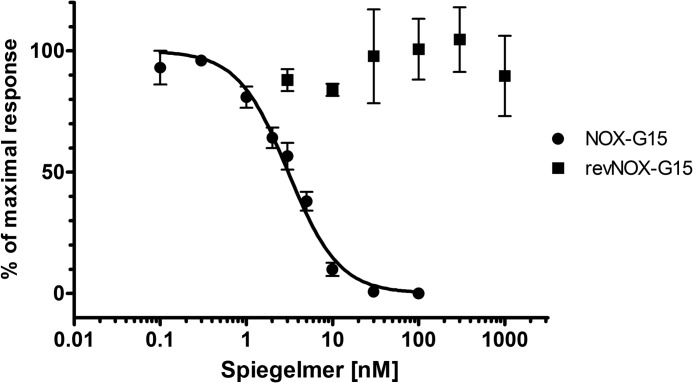
**Inhibition of glucagon-induced production of cAMP by NOX-G15.** CHO-K1 cells expressing the human glucagon receptor were incubated with 0.5 nm glucagon and increasing concentrations of NOX-G15 and the reverse control Spiegelmer, revNOX-G15. The amounts of glucagon-induced cAMP were normalized to the largest value of each data set and depicted as percent activity against Spiegelmer concentration. Data are mean ± S.E. of nine independent experiments for NOX-G15 and five independent experiments for revNOX-G15.

To demonstrate *in vitro* efficacy of NOX-G15, CHO-K1 cells expressing the human glucagon receptor were exposed to 0.5 nm glucagon and increasing concentrations of NOX-G15. NOX-G15 inhibited 50% of the glucagon-induced cAMP generation at a concentration of 3.07 nm (95% confidence interval: 2.73–3.46 nm, *n* = 9). revNOX-G15 was used as a control for unspecific binding. As expected, revNOX-G15 did not interfere with glucagon signaling (*n* = 3) (Fig. 5).

##### Pharmacokinetics in Mice

**FIGURE 6.**
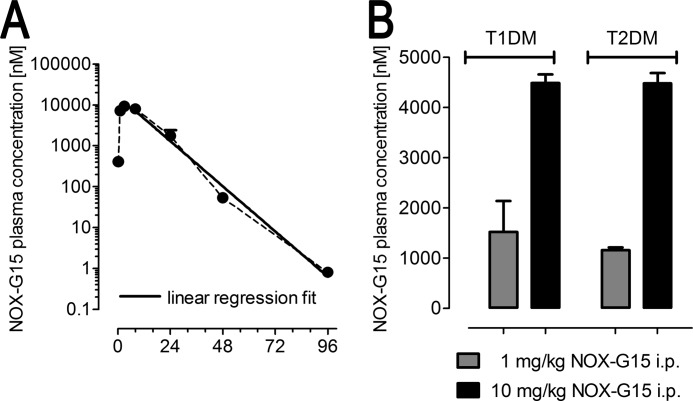
**Spiegelmer levels in mouse plasma.** *A,* pharmacokinetic analysis of NOX-G15 after intraperitoneal injection in mice. *B,* quantification of NOX-G15 in the terminal plasma after the ipGTT. NOX-G15 plasma levels were determined by Biacore measurement using an immobilized hybridization l-DNA probe being complementary to the 3′-end of NOX-G15 (*n* = 4 per group and time point).

Plasma pharmacokinetics after intraperitoneal injection of NOX-G15 were tested in mice (10 min to 96 h) to secure appropriate dose levels throughout ipGTTs (90 to 210 min after substance administration). 10 mg/kg led to plasma levels of 400 nm at 10 min post-administration, rising to a plateau from 1 to 8 h (*c*_max_ at 3 h = 9,600 nm). Plasma levels then declined with a half-life of 6.5 h (Fig. 6*A*).

##### Efficacy of NOX-G15 in Experimentally Induced T1DM and T2DM

**FIGURE 7.**
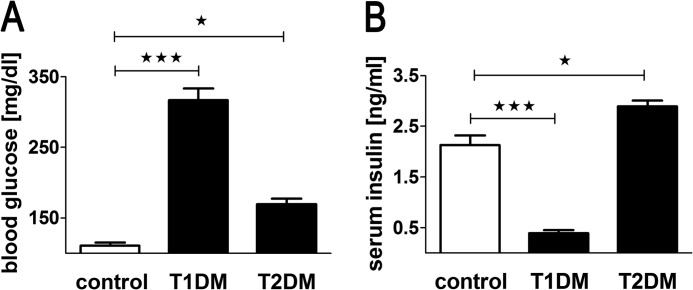
**Measures of diabetes induction after a 2.5-h fasting period.** *A*, blood glucose before test substance application and ipGTT. *B*, serum insulin levels, determined in separate groups (mean ± S.E.). *, *p* < 0.05; **, *p* < 0.01; ***, *p* < 0.001.

**FIGURE 8.**
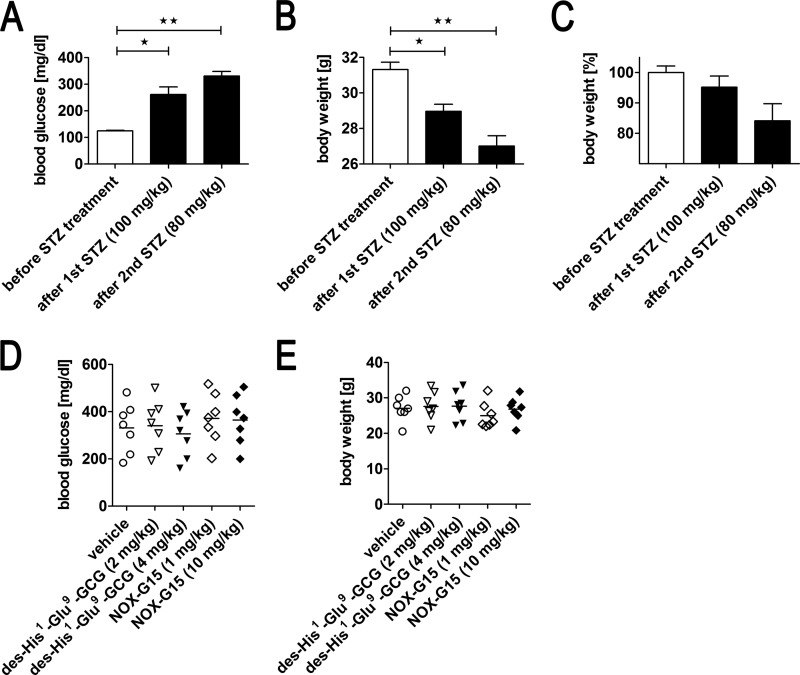
**Determination of T1DM status and group randomization.** *A,* blood glucose, *B,* body weight, and *C,* changes of body weight before STZ treatment, after the first STZ (100 mg/kg) injection and after the second STZ (80 mg/kg) administration 1 day before ipGTT. Data were analyzed using one-way ANOVA and Tukey post-test: *, *p* < 0.05; **, *p* < 0.01 (mean ± S.E.). *D,* blood glucose, and *E,* body weight distribution between animal groups with experimentally induced T1DM after randomization 1 day before ipGTT (means are indicated; *n* = 7 per group).

**FIGURE 9.**
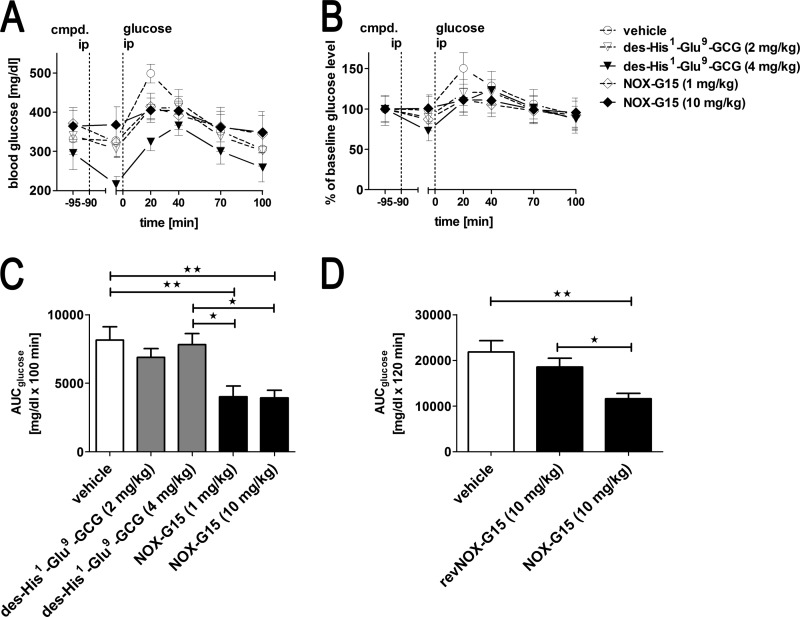
**Blood glucose during ipGTT in T1DM mice.** *A,* absolute blood glucose; *B,* percent of baseline glucose during ipGTT. *C,* blood glucose AUC (0–100 min) individually calculated for each animal; *n* = 7 per group. *D,* blood glucose AUC (0–120 min) in a confirmatory study including the non-functional Spiegelmer revNOX-G15; *n* = 8 per group. Baseline for AUC calculation was set to the glucose concentration at time −5 min. Mean ± S.E. Data were analyzed using one-way ANOVA and Tukey post-test: *, *p* < 0.05; **, *p* < 0.01.

T1DM was induced by two doses of STZ leading to incrementally increasing blood glucose and decreasing serum insulin and body weight (Figs. 7, *A* and *B*, and 8, *A–C*). The inclusion criteria allowed randomization of 35 animals (7 per group), which were assigned to the groups based on blood glucose and body weight (Fig. 8). The diabetic mice (T1DM) presented with blood glucose levels between 300 and 400 mg/dl after the 2.5-h fasting interval (Figs. 7, 8, and 9*A*). NOX-G15 did not significantly influence basal blood glucose levels (−95 min to −5 min). The peptidyl glucagon receptor antagonist des-His^1^-Glu^9^-GCG (28, 29) only reduced fasting blood glucose levels markedly in the high dose group (4 mg/kg) in T1DM (Fig. 9, *A* and *B*).

During ipGTT blood glucose peaked highest in the vehicle-treated group (499 ± 24 mg/dl) 20 min after the intraperitoneal glucose injection (2 g/kg body weight). Mice injected with des-His^1^-Glu^9^-GCG at both doses (2 and 4 mg/kg) showed a trend toward the attenuated increase in blood glucose as compared with vehicle-treated animals (Fig. 9, *A* and *B*). Analysis of the area under the curve revealed no significant effect of des-His^1^-Glu^9^-GCG *versus* vehicle (Fig. 9*C*). In contrast, both animal groups treated with NOX-G15 Spiegelmer had both a lower peak glucose concentration than vehicle and a statistically significant lower AUC than vehicle or glucagon receptor-treated animals (Fig. 9, *A–C*). In a subsequent experiment, revNOX-G15 (10 mg/kg, corresponding to the higher dose of NOX-G15) was included to verify that the beneficial effect of NOX-G15 was indeed glucagon-specific and not a matter of unspecific interactions potentially induced by any other Spiegelmer property, *e.g.* its polyanionic nature. 24 animals could be included and were randomized to three treatment groups (8 per group) based on body weight and blood glucose (data not shown). Although NOX-G15 again significantly reduced the glucose AUC, revNOX-G15 did not have a significant effect if compared with vehicle (Fig. 9*D*).

**FIGURE 10.**
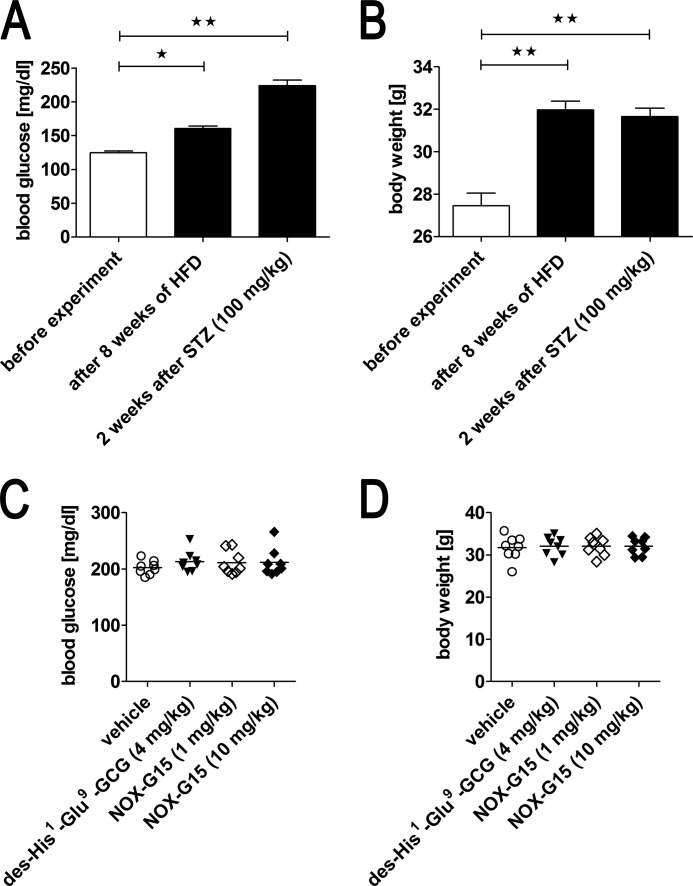
**Changes of blood glucose concentration and body weight during the course of T2DM induction and group randomization.** *A,* blood glucose, and *B,* body weight were determined before the experiment, after 8 weeks of high-fat diet, and 2 weeks after STZ (100 mg/kg) administration (1 day before ipGTT). Data were analyzed using one-way ANOVA and Tukey post-test: *, *p* < 0.05; **, *p* < 0.01 (mean ± S.E.). *C,* blood glucose, and *D,* body weight distribution between animal groups with experimentally induced T2DM after randomization 1 day before ipGTT (means are indicated, *n* = 8–9 per group).

**FIGURE 11.**
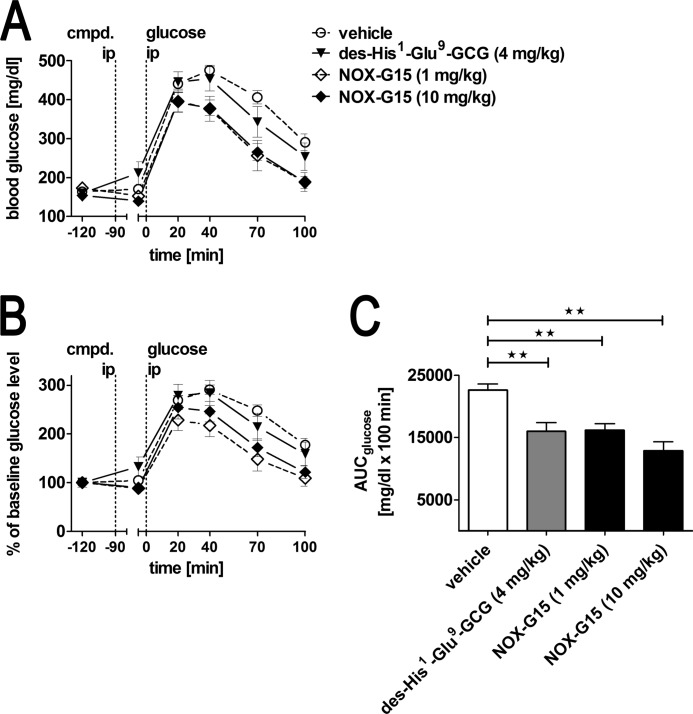
**Blood glucose during ipGTT in T2DM mice.** *A,* absolute blood glucose; *B,* percent of baseline blood glucose during ipGTT. *C*, blood glucose AUC individually calculated for each animal. Baseline for AUC calculation was set to the glucose concentration at time −5 min. Mean ± S.E.; *n* = 8–9 animals per group. Data were analyzed using one-way ANOVA and Tukey post-test: **, *p* < 0.01.

T2DM was induced by HFD over 10 weeks and a single STZ injection after week 8. Both blood glucose and body weight increased during this time (Fig. 10). The inclusion criteria allowed inclusion of 35 animals (8–9 per group), which were subsequently randomized based again on blood glucose and body weight (Fig. 10, *C* and *D*). On the day of the ipGTT T2DM mice presented with elevated glucose concentrations of 170 ± 8 mg/dl (mean ± S.E.) after the 2.5-h fasting interval (Fig. 11*A*). In comparison, non-diabetic mice had a basal glucose concentration of 111 ± 5 mg/dl (Fig. 7). NOX-G15 administration did not induce a significant decrease of fasting blood glucose 85 min post-substance administration.

40 min after the intraperitoneal glucose injection, blood glucose peaked highest in the vehicle-treated group (476 ± 12 mg/dl). After 100 min, blood glucose was not fully restored to pre-dose values in the vehicle-treated group (290 ± 21 *versus* 164 ± 4 mg/dl). The glucagon receptor antagonist des-His^1^-Glu^9^-glucagon (4 mg/kg) was statistically ineffective at reducing peak blood glucose compared with vehicle (454 ± 31 *versus* 476 ± 12 mg/dl) in the ipGTT; nevertheless, a trend toward a faster recovery of blood glucose to normal levels was observed resulting in a significantly lower AUC compared with vehicle (Fig. 11, *A–C*).

During the ipGTT, type 2 diabetic animals treated with the low or the high dose of NOX-G15 had significantly lower peak blood glucose concentrations than vehicle-treated control diabetic mice (396 ± 27 and 393 ± 19 mg/dl, respectively, *versus* 476 ± 12 mg/dl) (Fig. 11, *A* and *B*). At the 40-min time point, elevated blood glucose concentrations started to fall, followed by a sustained rapid decline with normalization after 100 min. Thus, both doses of NOX-G15 showed comparable efficacy at lowering blood glucose concentrations in ipGTT. Both Spiegelmer doses also led to statistically significant lowering of blood glucose excursions, as judged by AUC in comparison to animals, which had received vehicle (Fig. 11*C*).

##### Quantification of NOX-G15 Plasma Levels in Terminal Plasma

The terminal plasma was analyzed from *n* = 4 animals of both studies (T1DM and T2DM) using a NOX-G15 capture probe on the Biacore system. In T1DM mice, intraperitoneal injection of 1 and 10 mg/kg NOX-G15 led to plasma levels of 1.7 ± 0.6 and 4.5 ± 0.2 μm, respectively, 190 min post-administration. In T2DM mice injected intraperitoneally with either 1 or 10 mg/kg of NOX-G15 plasma levels after 210 min were 1.2 ± 0.1 and 4.5 ± 0.2 μm, respectively (Fig. 6*B*).

## DISCUSSION

Taking into consideration that hyperglucagonemia considerably contributes to hyperglycemia in both T1DM and T2DM, lowering of glucagon secretion and/or interference with glucagon action on target tissues represents a therapeutic principle to improve glucose control that is currently not used in clinical practice. In our study we report the identification of a novel therapeutic modality that can counteract glucagon action through high affinity binding. This compound, NOX-G15, is a structured, mirror-image oligonucleotide (a so-called Spiegelmer), conjugated to a 40-kDa polyethylene glycol moiety that can interact with and block glucagon in a manner conceptually similar to a monoclonal antibody.

The compound was identified using an evolutionary screening technique to identify biostable aptamers (13). At first, aptamers were identified to bind the enantiomer of the natural target (d-glucagon). Then, the aptamer sequences were synthesized using mirror-image nucleotide to give the Spiegelmer that binds with equal affinity to the natural target (l-glucagon). Glucagon turned out to be a difficult target for the identification of high-affinity nucleic acid-based binders. A reason may be the high entropic cost of forming a, usually rigid, complex from two highly flexible partners so that the selection/evolution of aptamers that have a rigid structure themselves was required. Eventually, glucagon-binding DNA oligonucleotides could be selected that were able to recognize glucagon with a dissociation constant (*K_d_*) of 172 nm. Because this *K_d_* was considered not sufficient for a drug approach, the binding was further tuned by exchanging single deoxynucleotides for corresponding ribonucleotides. Indeed, six positions in the glucagon-binding DNA oligonucleotide were identified that significantly improved the affinity when changed to RNA building blocks. In fact, the combination of these six RNA positions even led to synergistic effects so that the affinity was improved by a factor of 27 to give a final *K_d_*_(comp)_ of approximately 6 nm. Thus, it was far more effective than the mutagenic PCR approach used in the higher *in vitro* selection rounds that only led to a 2-fold affinity gain. Furthermore, the mixed DNA/RNA oligonucleotide could be truncated to a minimal binding motif of 39 nucleotides without loss of affinity. Interestingly four of the six substituted deoxyribonucleotides are G nucleotides located in the G-rich pattern, potentially forming a G-quadruplex. The fact that DNA-RNA nucleotide exchanges introduce conformational changes in a G-quadruplex structure has been shown before (31, 32). However, to our knowledge this is the first report demonstrating that affinity could markedly be increased by the introduction of discrete 2′-oxygens into the sugar moieties of a DNA aptamer. Whether the increased affinity results from increased duplex melting temperature (stability) (33), increased helix rigidity (34, 35), altered helix torsion angles (36), or the introduction of dipole forces and potential hydrogen bond donors that are beneficial for the target interaction at certain positions, can probably only be answered by determining the co-crystal structure of glucagon and the pure DNA Spiegelmer in comparison with the structure of glucagon and the mixed DNA/RNA Spiegelmer.

After defining the final 39-nucleotide-long oligonucleotide sequence, the 5′ terminus was modified with a 40-kDa PEG moiety to prolong plasma half-life of the Spiegelmer compound (22) for *in vivo* efficacy studies. This site-directed PEGylation of the Spiegelmer that gave the final Spiegelmer NOX-G15 did not impair binding affinity to and inhibition of its target *in vitro*. In a competitive Biacore study NOX-G15 was found to selectively bind glucagon with a *K_d_* of 3 nm and also OXM, which fully contains the glucagon sequence. OXM, which is secreted from intestinal l-cells after food ingestion is a dual agonist for both the GLP-1 and glucagon receptors. It may thus represent a two-edged sword in diabetes because GLP-1 signaling is believed to be beneficial. Metabolic activities of pharmacological doses of OXM were studied in humans and rodents. Administration of OXM resulted in weight reduction, increased energy expenditure, improvement of glucose tolerance, inhibition of gastric acid, and exocrine pancreas secretion (reviewed in Ref. 37). However, plasma levels of OXM are low, allowing the authors of the extensive review to conclude that the physiological significance of OXM is a matter to debate.

Finally, for *in vivo* proof of concept, the effect of single doses of NOX-G15 was evaluated in glucose tolerance tests in murine models of diabetes. In comparison to the peptidyl glucagon receptor antagonist des-His^1^-Glu^9^-glucagon (28), the anti-glucagon Spiegelmer NOX-G15 was able to lower glucose excursion in ipGTT (1.5 h post substance administration) in both animal models of T1DM and T2DM, whereas the glucagon receptor antagonist des-His^1^-Glu^9^-glucagon only proved efficacious in T2DM.

We assume that des-His^1^-Glu^9^-glucagon may have an unfavorable plasma elimination rate, which is most likely responsible for the weak improvement of glucose excursions, particularly in the severely hyperglycemic T1DM mice. Importantly, NOX-G15 did not cause hypoglycemia after a fasting period of 2.5 h. A longer period of fasting in diabetic animals was not evaluated in this study and may be specifically addressed in follow-up studies. Hypoglycemia in therapy of diabetes mellitus is a major concern, but even overnight fasting of mice with genetically deleted glucagon receptor only led to marginally lower blood glucose levels as compared with wild type animals, without occurrence of hypoglycemia. Moreover, the glucagon receptor-deficient mice had been found to have increased glucose tolerance (38). In a study with diabetic obese db/db mice, 83% reduction of glucagon receptor expression by antisense oligonucleotides did not induce hypoglycemia despite injection of exogenous insulin (39). Furthermore, in studies utilizing alternative approaches to block glucagon signaling with specific anti-glucagon or anti-glucagon receptor antibodies in mice and cynomolgus monkeys, hypoglycemia was not observed (40–42).

Comparable with the results of studies performed in mice lacking glucagon receptors (38), both doses of NOX-G15 effectively reduced the peak of glucose increase in ipGTT in both T1DM and T2DM animal models, allowing blood glucose to decline to pre-test levels after 70 or 100 min. The similar efficacy of both doses suggests that a saturation of the possible pharmacologic effect has been reached and that lower doses may also be effective. This is quite likely in the light of the inhibitory constant of NOX-G15 that is more than 400-fold below the lowest NOX-G15 plasma concentration measured at the end of the experiment. The pharmacokinetic analysis suggests that daily dosing of NOX-G15 would be sufficient to maintain stable plasma levels in murine multidose studies. Whether chronic dosing may also lead to improved basal glucose levels, which had been observed in glucagon receptor-deficient, STZ-treated T1DM mice (6) and after 14 days of anti-glucagon antibody treatment in diabetic ob/ob mice (41), will be the subject of further investigation.

In summary this is the first report describing the generation of a glucagon-binding synthetic oligonucleotide that interferes with endogenous glucagon and acutely improves glucose tolerance. Only two compounds (Lilly's receptor antagonist and Isis' above mentioned receptor antisense oligodeoxyribonucleotide) are active in clinical testing despite the long-standing knowledge of the potential benefits of glucagon inhibition in diabetes and broad efforts to identify potent glucagon receptor antagonists. NOX-G15 may offer an alternative by selectively interfering with glucagon to alleviate glycemic excursions in diabetes mellitus.

## Footnotes

3 Spiegelmer® is a registered trademark of NOXXON Pharma AG.

## Footnotes

2 The abbreviations used are:

T2DM: type 2 diabetes mellitus
T1DM: type 1 diabetes mellitus
STZ: streptozotocin
GLP: glucagon-like peptide
OXM: oxyntomodulin
GIP: glucose-induced insulinotropic peptide
prepro-VIP: prepro-vasoactive intestinal peptide
ipGTT: intraperitoneal glucose tolerance test
PEG: polyethylene glycol
HFD: high-fat diet
ANOVA: analysis of variance
nt: nucleotide(s)
AUC: area under the curve.
